# Guided Bone Regeneration Using Collagen Scaffolds, Growth Factors, and Periodontal Ligament Stem Cells for Treatment of Peri-Implant Bone Defects *In Vivo*

**DOI:** 10.1155/2017/3548435

**Published:** 2017-08-16

**Authors:** Peer W. Kämmerer, Malte Scholz, Maria Baudisch, Jan Liese, Katharina Wegner, Bernhard Frerich, Hermann Lang

**Affiliations:** ^1^Department of Oral and Maxillofacial Surgery, Facial Plastic Surgery, University Medical Centre Rostock, Schillingallee 35, 18057 Rostock, Germany; ^2^Department of Operative Dentistry and Periodontology, University Medical Centre Rostock, Strempelstraße 13, 18057 Rostock, Germany

## Abstract

**Introduction:**

The aim of the study was an evaluation of different approaches for guided bone regeneration (GBR) of peri-implant defects in an *in vivo* animal model.

**Materials and Methods:**

In minipigs (*n* = 15), peri-implant defects around calcium phosphate- (CaP-; *n* = 46) coated implants were created and randomly filled with (1) blank, (2) collagen/hydroxylapatite/*β*-tricalcium phosphate scaffold (CHT), (3) CHT + growth factor cocktail (GFC), (4) jellyfish collagen matrix, (5) jellyfish collagen matrix + GFC, (6) collagen powder, and (7) collagen powder + periodontal ligament stem cells (PDLSC). Additional collagen membranes were used for coverage of the defects. After 120 days of healing, bone growth was evaluated histologically (bone to implant contact (BIC;%)), vertical bone apposition (VBA; mm), and new bone height (NBH; %).

**Results:**

In all groups, new bone formation was seen. Though, when compared to the blank group, no significant differences were detected for all parameters. BIC and NBH in the group with collagen matrix as well as the group with the collagen matrix + GFC were significantly less when compared to the collagen powder group (all: *p* < 0.003).

**Conclusion:**

GBR procedures, in combination with CaP-coated implants, will lead to an enhancement of peri-implant bone growth. There was no additional significant enhancement of osseous regeneration when using GFC or PDLSC.

## 1. Introduction

Dental implants made of titanium and its alloys show high long-term survival and success rates [1, 2]. Though, implant failure exists that has been mainly attributed to inflammatory processes of the peri-implant tissues [3], mostly due to accumulation of plaque around the mucosal margins of the implants [4]. These processes include peri-implant mucositis and peri-implantitis being the two main disease entities. Whereas peri-implant mucositis is defined as inflammation in the mucosa at an implant with no signs of loss of supporting bone, peri-implantitis combines inflammation in the mucosa and respective bone loss past normal biological remodeling [5]. It was reported that the prevalence of peri-implant mucositis is 43% whereas 22% of the implants show peri-implantitis [6]. Nevertheless, these numbers should be handled with care due to different case definitions, diagnostic methods, as well as different thresholds for probing depth, and bone loss [7].

Even despite adequate peri-implant maintenance therapy, some patients will develop these soft and hard tissue complications [8]. Untreated peri-implantitis is critical and may finally lead to loss of the affected implant [9]; therefore, an intervention should be carried out before substantial amounts of supporting bone are lost. Before treatment of peri-implantitis, iatrogenic factors such as remnants of cement, malpositioning of the implant, inadequate restoration-abutment sealing, overcontouring of the reconstruction, and other technical complications should be ruled out [7]. After excluding these parameters, specific treatment modalities for peri-implantitis include cleaning via a variety of different techniques, using of antibiotics, or even removing of the implants. At the moment, there is no firm or specific evidence-based recommendation for a specific therapy [10] as neither one of the cleaning methods nor the antiseptic/antibiotic therapy has proven 100% success.

Mechanical cleaning seems to be a prerequisite but has shown to be insufficient for promotion of osseous regeneration [11] that is an important outcome criterion with an immediate effect on the implant surface decontamination protocol [12]. Additional guided bone regeneration (GBR) techniques using different biomaterials have been advocated for management of peri-implant defects [13–16]. For example, collagen matrices alone may enhance soft- and hard-tissue regeneration [17]. Furthermore, growth factors in combination with carrier materials such as collagen or bone substitute materials may modulate and enhance cellular proliferation leading to a better regrowth of bone [18, 19]. Also, periodontal ligament stem cells (PDLSC) obtained from oral tissues in combination with scaffold systems and growth factors have shown to have an osseous regeneration potential [20, 21].

Up to date, no predictable regenerative protocol for regeneration of peri-implant defects has been established. Therefore, the aim of the *in vivo* study was to evaluate different approaches for regeneration of osseous peri-implant defects using different collagen carriers alone as well as in combination with growth factors and PDLSC.

## 2. Materials and Methods

### 2.1. Animals

The study was performed with 15 female Göttingen miniature pigs (22 ± 3 months, 35 ± 11 kg). The pigs were reared under conventional conditions at the Leibniz Institute for Farm Animal Biology (18196 Dummerstorf, Mecklenburg-Western Pomerania) with free access to water and soft diet. The pigs were labelled with earmarks. The whole study was monitored by the local authority and permitted according to the German animal protection act (German Decree on the Reporting of Laboratory Animals 7221.3-1.1-075/11, Regional Authority for Agriculture, Food Safety and Fisheries, State of Mecklenburg-Western Pomerania, Germany).

### 2.2. Surgical Procedure

#### 2.2.1. Anesthesia

The study was performed similarly as previously described by our group [22]. All surgical interventions were performed under sterile conditions and general anesthesia. Preoperatively, each animal received 1.5 ml midazolam intramuscularly (Sanochemia Pharmazeutika AG, Neufeld, Austria) and 10% solution of ketamine (Sanochemia Pharmazeutika AG, Neufeld, Austria). Further intravenous injection was carried out with 0.25–0.4 ml pancuronium (2 mg/ml, Organon Teknika, Eppelheim, Germany) for muscle relaxation. The initiation of oral intubation anesthesia was performed with fentanyl (0.5–0.8 ml/min, Janssen-Cilag, Neuss, Germany) and sustained with 1.5% isoflurane (AbbVie AG, Baar, Switzerland) together with an additional administration of oxygen (1.5 l/min). Immediately after sedation, the perioral hair region was shaved and disinfected with povidone iodine solution (Betaisodona®, Munidpharma GmbH, Limburg an der Lahn, Germany). Subsequently, the region of operative interest was locally anesthetized with 4% articaine (1 : 100.000, 2 ml, Sanofi Deutschland GmbH, Frankfurt, Germany). During surgery, the miniature pigs received intravenous antibiotics (ampicillin/sulbactam, 1000 mg/500 mg; Hexal AG, Holzkirchen, Germany). Postoperatively, each animal received analgesia treatment (15 mg/ml Metacam® suspension, dose: 2.7 ml/100 kg body weight, Boehringer Ingelheim Vetmedica GmbH, Ingelheim am Rhein, Germany) as well as oral antibiosis (Synulox®, 250 mg, 2 × 1, Pfizer AG, New York, USA).

### 2.3. Extraction and Tissue Removal

In both quadrants of the lower jaw, the 1st and 2nd premolars were extracted. The extracted teeth were cleaned and rinsed with different volumes of phosphate buffered saline (20 ml–50 ml) complemented with antibiotics (200 *μ*l 1 × penicillin/streptomycin, Gibco, Grand Island, NY, USA) in order to diminish bacterial infection. Afterwards, the samples were prepared for transportation in 50 ml tubes containing DMEM-F_12_ media (Gibco, Grand Island, NY, USA) with 2% penicillin/streptomycin (Gibco, Grand Island, NY, USA) at a constant temperature of 4°C.

### 2.4. Isolation and Cultivation of the PDLSC out of the Obtained Tissue

The isolation of the porcine periodontal ligament stem cells (PDLSC) followed the protocol of Haddouti et al. [23]. First, the periodontal ligament was isolated from the roots of the extracted teeth and bruised to small pieces under aseptic conditions. The periodontal ligament was rinsed with 5 ml of DMEM-F_12_ (Gibco, Grand Island, NY, USA) with 2.5 mg/ml dispase (Sigma-Aldrich, St. Louis, USA) and incubated for 1-2 h at 37°C and 5% CO_2_. After centrifugation at 400 ×g for 4 min at 4°C, the supernatant was discarded. The pellets were dissolved in 3 ml cell culture medium (DMEM-F_12_ containing 10% fetal bovine serum; Biochrom, Berlin, Germany) and transferred into cell culture flasks (Cellstar®, Greiner-bio one GmbH, Kremsmünster, Austria) including 1% penicillin/streptomycin (Gibco, Grand Island, NY, USA). The flasks were incubated at 37°C and 5% CO_2_ for 24 h. After 24 h, the cell culture was controlled via light microscopy for bacterial contamination. Floating cells were removed, and the cell culture flask was refilled with 20 ml fresh cell culture medium. After an incubation time of 1-2 weeks at 37°C and 5% CO_2_ and regular medium change (every 3 days), the cells grew adherent to plastic. After confluent growing, the progenitor cells were passaged by the use of 2 ml trypsin (Gibco, Grand Island, NY, USA). The cell culture medium was discarded, trypsin added, and incubated for 5 min at 37°C until the cells could be removed from the bottom of the flask. The characterization of PDLSC was conducted via flow cytometry, and the surface markers CD 29, CD 44, and CD 90 were verified.

**Figure 1:**
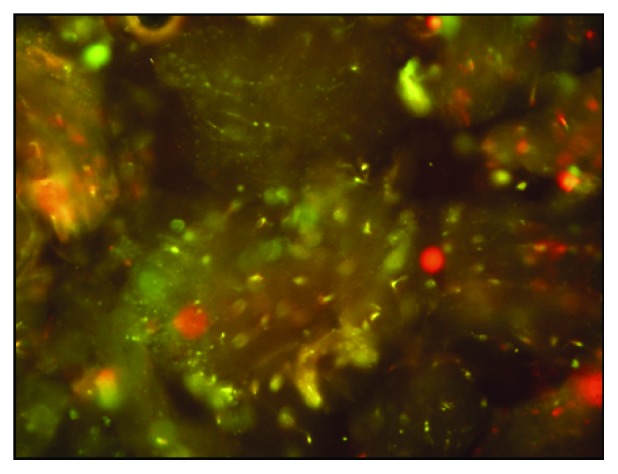
Live-death staining of PDLSC located in the buffered collagen powder 24 h after incubation. Most cells are alive (colored in green).

For application of the cells, 10^6^ PDLSC of the 3rd or 4th passage were combined with a collagen powder (fibrillary collagen type I, III, and V from bovine tissue; MedSkin Solutions, Dr. Suwelack AG, Billerbeck, Germany). In brief, after buffering the acidic pH (pH ~4-5) of the collagen powder to a neutral pH value (pH ~7), PDLSC were transferred to the powder [22]. After 24 h, prior implantation, a life-death-stain was performed in order to evaluate the survival of the stem cells and to ensure that vial cells were used (Figure 1).

### 2.5. Implantation and Creation of Circular Defects as well as Insertion of Different Materials

**Figure 2:**
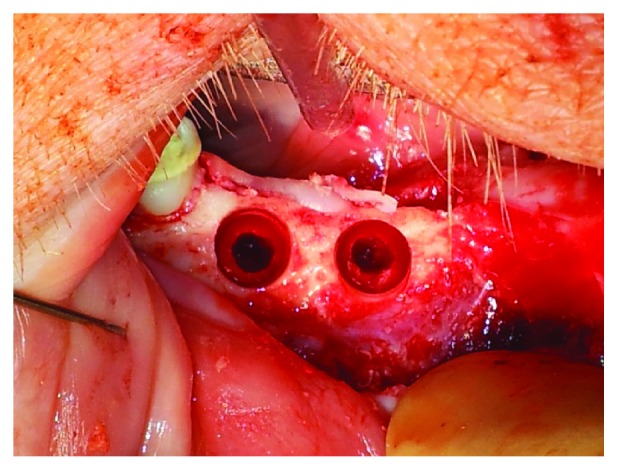
Clinical site showing the prepared implant beds as well as the circumferential defects.

**Figure 3:**
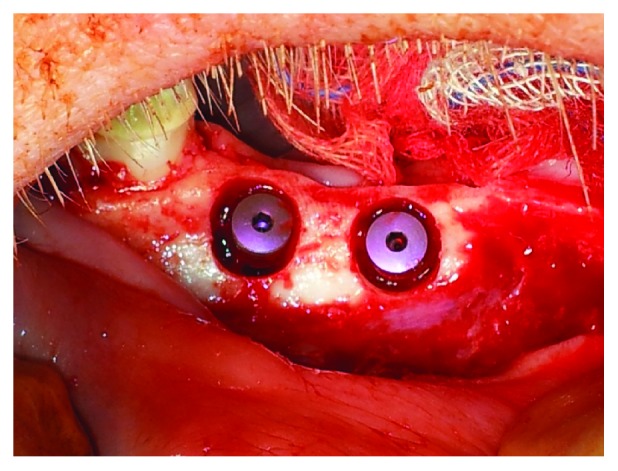
Clinical site showing the inserted dental implants as well as the circumferential defects.

Three months after healing, implants were inserted at the former position of the extracted premolars. First, a crestal mucoperiosteal flap was prepared. The flap was mobilized, and the implant drilling procedures were performed following the manufacturer's instructions using configured drills. In order to create the circular peri-implant bone defects, the upper 5 mm of the total depth of 12 mm was widened to 7 mm (Figure 2). In each semi-mandible, 1-2 4.3 × 12 mm implants (total *n* = 46) were inserted (Alphatech® Tube-line BONITex®, FMZ GmbH, Rostock, Germany) (Figure 3). BONITex combines sandblasting, acid-etched implants with a thin, quick absorbable calcium phosphate (CaP) layer [24–26]. All circular defects were randomly filled with different collagen materials solely or in combination with growth factors or PDLSC by the use of a sterile spatula. Randomization was conducted using sealed envelopes with the respective group number that was opened before each surgical procedure.

In group I, no materials were inserted (blank group). In group II, the defect was filled with a collagen/hydroxylapatite/*β*-tricalcium phosphate scaffold (CHT; 1% collagen and 60 : 40 mixtures of hydroxylapatite/*β*-tricalcium phosphate; BONITmatrix®, DOT GmbH, Rostock, Germany). In group III, CHT together with 0.15 ml of a growth factor cocktail (GFC) obtained from gamma sterilized human platelet lyophilisate and dissolved in 0.9% sodium chloride solution up to a concentration of 2 mg/ml was used (injected amount: 0.3 mg mixture of growth factors (178.7 pg VEGF, 64.8 pg d-FGF, 90.2 pg IGF-1, and 52023.8 pg TGF-*β*1); DOT GmbH, Rostock, Germany). In groups IV and V, a matrix consisting of jellyfish collagen (*Rhopilema* sp.) without and with the respective GFC was used. In group VI, the collagen powder (fibrillary collagen types I, III, and V from bovine tissue; MedSkin Solutions, Dr. Suwelack AG, Billerbeck, Germany) was inserted into the defects. For group VII, PDLSC incubated into the collagen powder were applied.

In brief, the different tested materials for regeneration of bony peri-implant defects were the following:

- group I: blank, blood coagulum (*n* = 6)
- group II: collagen/hydroxylapatite/*β*-tricalcium phosphate scaffold (CHT; *n* = 8)
- group III: CHT plus growth factor cocktail (GFC; *n* = 7)
- group IV: collagen matrix (*n* = 6)
- group V: collagen matrix plus GFC (*n* = 5)
- group VI: collagen powder (*n* = 6)
- group VII: collagen powder plus PDLSC (*n* = 8)

All defects were additionally covered with a semipermeable membrane (35 × 45 mm, Angiopore®, Bredent Medical, Senden, Germany) for GBR purposes, and the mucoperiosteal flap was replaced and fixed with absorbable sutures (Vicryl® 3-0, Ethicon, Johnson & Johnson Medical GmbH, Norderstedt, Germany).

### 2.6. Sequential Labelling with Polychrome Dyes

Finally, the polychrome sequential labelling for histological evaluation of the new bone formation and remodeling processes occurred. All miniature pigs were injected intravenously (10 ml/min) with three different fluorochromes xylenol orange (6%, 2–5 g/animal), calcein green (1%, 0.8–1.5 g/animal), and alizarine complexone (3%, 1–1.5 g/animal) 14, 28, and 84 days after implantation.

### 2.7. Preparation of Histological Sections

120 days after implantation, the animals were sacrificed under general anesthesia by the administration of an overdose of thiopental (Ospedalia AG, Hünenburg, Switzerland). After intubation, the preparation and catheterization of Vv. jugulares externae and Aa. carotes externae were conducted. Fixation of the oral tissues followed through the carotid arteries by dispensation of 10% formaldehyde (Helm Austria GmbH, Wien, Austria), and the mandibles of the miniature pigs were exarticulated and carved into segments. The saw cuts were fixed in 4% formalin (Formafix®, Global Technologies Ltd., Düsseldorf, Germany) for 7 days and dehydrated for 14 days with increasing concentrations of alcohol (70%, 80%, 96%, and 100%). Over a period of 28 days, the sections were block-embedded in PMMA (Technovit® 7200 VLC, Heraeus Kulzer GmbH, Hanau, Germany). The specimens were ground in sagittal direction and cut with a microtom (EXAKT Advanced Technologies GmbH, Norderstedt, Germany) into 250 *μ*m thick sections. The sections were further reduced to 15 *μ*m, polished, and stained with toluidine blue as described before [27].

### 2.8. Histomorphometric Analysis

**Figure 4:**
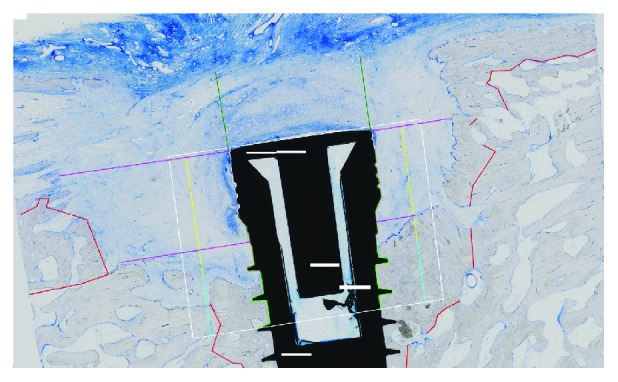
Histological specimen (implant with collagen/hydroxylapatite/*β*-tricalcium phosphate scaffold; toluidine blue, original magnification ×10) showing the calculated parameters: BIC (green), VBA (yellow), and NBH (turquoise).

The histological evaluation was performed with an optical light microscope (Carl Zeiss, Axio Imager M2, Jena, Germany) in an observer-blinded manner. The sections were scanned with a digital microscope camera (Axiocam MRC5, Carl Zeiss, Jena, Germany) and analyzed with the help of the program AxioVision SE64 Rel. 4.8 (Carl Zeiss, Jena, Germany). In every histological sample, a region of interest (ROI) was marked in a dimension of 7 × 5 mm with the implant in the middle and the former defect at the upper part. All measurements were carried out within the ROI. Tissues with high formation rates of new bone accumulated the fluorescent dye and could be observed with a fluorescence microscope (Carl Zeiss, Axiovert 40 CFL, Axiocam MRC5, Jena, Germany) at wavelengths from 490 to 520 nm (stimulating wavelength for calcein green). In every sample, the frontiers of new bone formation were marked with red lines and the areas were determined by the use of the software ImageJ. The following parameters were measured on both sides of the implants, and mean values were calculated:

- Bone to implant contact (BIC; %) describes the length of the implant surface within the ROI that was in direct apposition of bone x100%. Mean values were created out of the values for the mesial and the distal sides.
- Vertical bone apposition (VBA; mm) describes the new formed bone in contact with the implant from implant shoulder to the first thread of the implant that is within the residual bone.
- New bone height (NBH; mm) describes the height of new bone formation within the defect (Figure 4).

### 2.9. Statistical Analysis

This *in vivo* study had a planned case number to be equal if not higher to similar studies comparing treatment of peri-implant bone defects [12, 28]. Mean, median values, as well as standard deviations of the three parameters, were calculated descriptively. The obtained consistent data were visualized via box plots. In the further explorative data analysis, Kolmogorov-Smirnov tests were employed in order to examine differences between the groups. In cases of *p* values < .05, Mann–Whitney *U* tests, and, in cases of *p* values > .05, Student's *t*-test for independent samples were employed. The (descriptive) significance level was set at *p* ≤ 0.05. All analyses were conducted using SPSS 24.0 for Mac (IBM, Armonk, NY, USA).

## 3. Results

### 3.1. Bone to Implant Contact (BIC; %)

**Figure 5:**
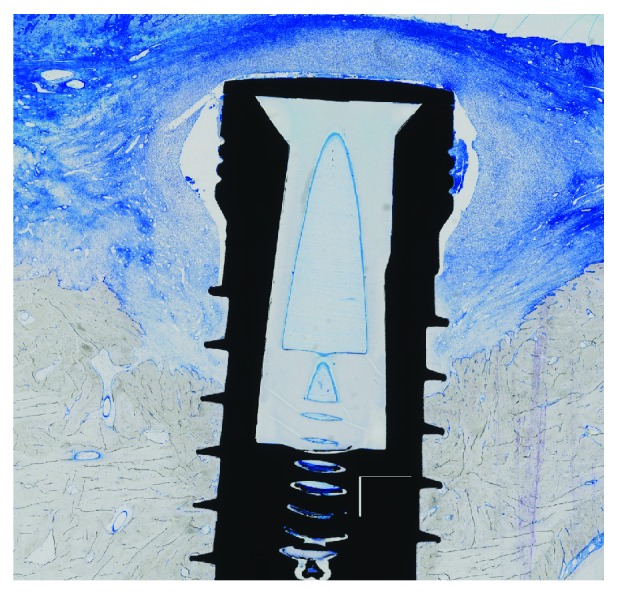
Histological specimen (implant with jellyfish collagen matrix; toluidine blue, original magnification ×10).

**Figure 6:**
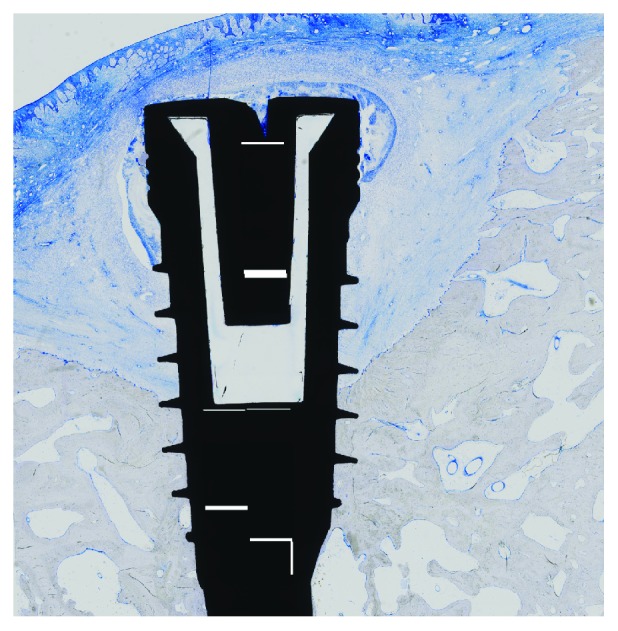
Histological specimen (implant with jellyfish collagen matrix + growth factor cocktail; toluidine blue, original magnification ×10).

**Figure 7:**
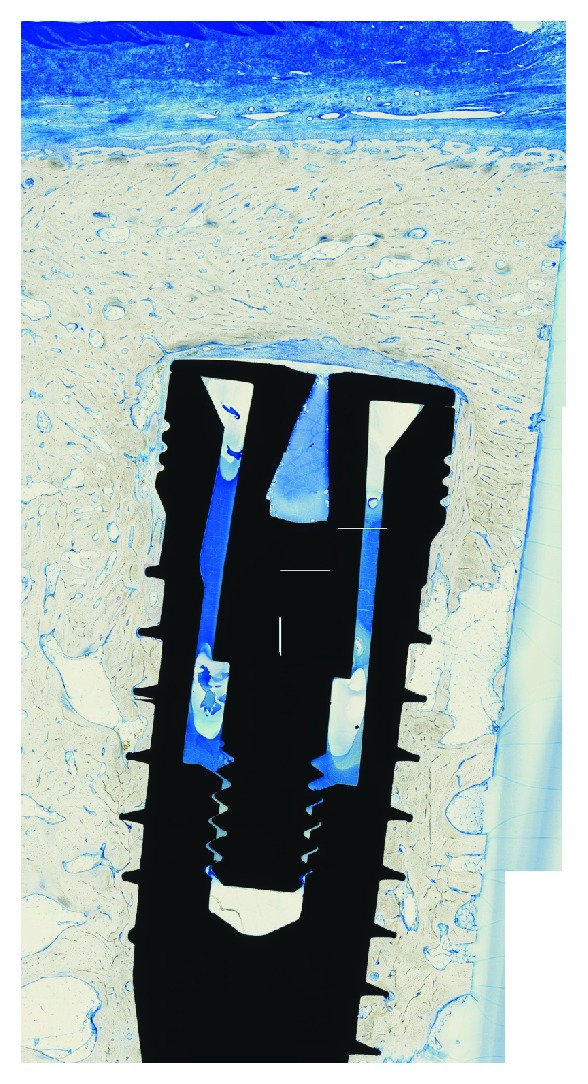
Histological specimen (implant with bovine collagen powder; toluidine blue, original magnification ×10).

**Figure 8:**
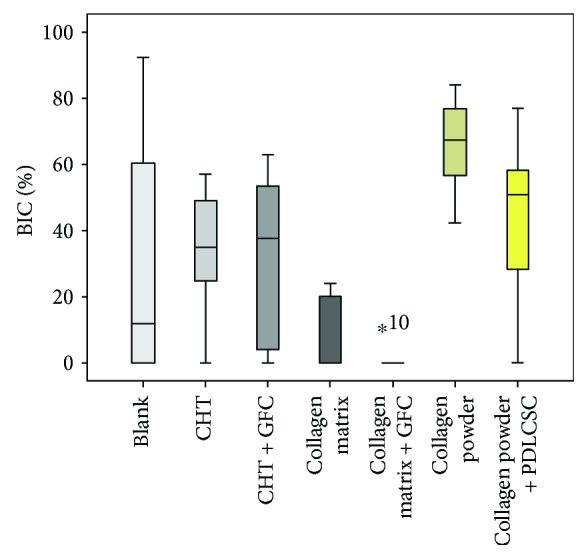
Boxplots showing the bone implant contact (BIC; %) when using different scaffolds (CHT = collagen/hydroxylapatite/*β*-tricalcium phosphate scaffold; GFC = growth factor cocktail; PDLSC = periodontal ligament stem cells). ^*^No mean value calculated, only one case was evaluable.

**Figure 9:**
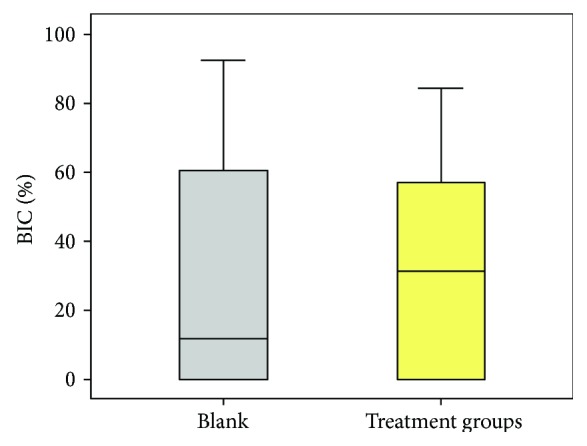
Boxplots comparing the bone implant contact (BIC; %) in the blank versus all treatment groups.

The highest BIC was seen in cases with collagen powder (mean value (MV) 65.8%, standard deviation (SD) 15.5) followed by collagen powder + PDLSC (MV 44%, SD 24.3%) and the CHT groups (MV 34.4%, SD 18.5%). Less BIC was calculated for the group with CHT + GFC (MV 30.7%, SD 27.7%) and the blank group (MV 29.4%, SD 38.9%). The least BIC values were seen in the collagen matrix (MV 7.3%, SD 11.5%) and the collagen matrix + GFC (MV 2%, SD 4.4%). BIC in the group with the collagen matrix (Figure 5) as well as the group with the collagen matrix + GFC (Figure 6) was significantly less when compared to the collagen powder group (*p* = 0.002 and *p* < 0.001; Figures 7 & 8). When comparing the blank group with all treatment groups (MV 29.4%, SD 38.9% versus MV 32.3%, SD 27.4%), no significant differences were seen (*p* = 0.821; Figure 9).

### 3.2. Vertical Bone Apposition (VBA; mm)

**Figure 10:**
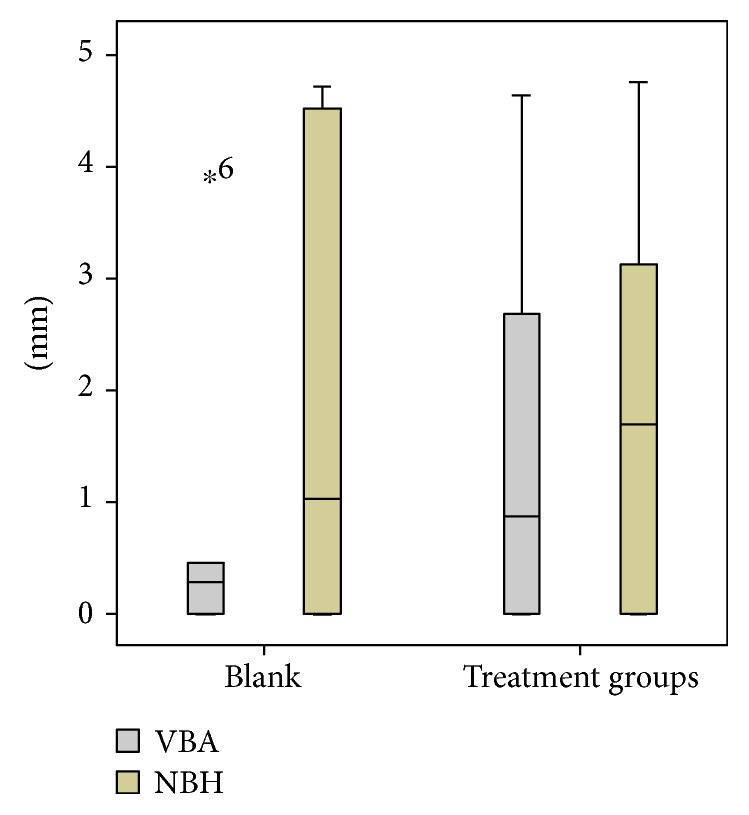
Boxplots comparing the vertical bone apposition (VBA; mm) and the new bone height (NBH; mm) in the blank versus all treatment groups. ^*^No mean value calculated, only one case was evaluable.

The highest VBA was seen in the group with CHT (MV 2.2 mm, SD 1.25 mm) followed by the group with collagen powder + PDLSC (MV 1.7 mm, SD 1.3 mm) and the group with CHT + GFC (MV 1.4 mm, SD 1.24 mm). The values in the other groups were similar (blank MV 0.93 mm, SD 1.67 mm; collagen matrix MV 1 mm, MV 2 mm; collagen matrix + GFC MV 0.93 mm, SD 2.1 mm; collagen powder MV 0.94 mm, SD 1.2 mm). Between the groups, no statistical significant differences were seen (all *p* > 0.05). The comparison between the blank group and all treatment groups did not show significant differences as well (MV 0.93 mm, SD 1.67 mm versus MV 1.4 mm, SD 1.4 mm; *p* = 0.499; Figure 10).

### 3.3. New Bone Height (NBH; mm)

**Figure 11:**
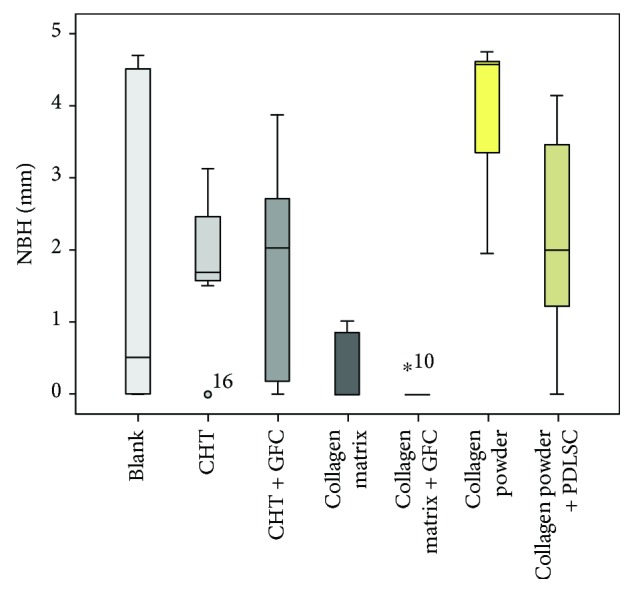
Boxplots showing the height of new bone formation within the former defect (NBH; mm) when using different scaffolds (CHT = collagen/hydroxylapatite/*β*-tricalcium phosphate scaffold; GFC = growth factor cocktail; PDLSC = periodontal ligament stem cells). ^*^No mean value calculated, only one case was evaluable.

Within the created defect, the highest rate of NBH was seen in the group using collagen powder (MV 3.96 mm, SD 1.1 mm) followed by collagen powder + PDLSC (MV 2.1 mm, SD 1.4 mm) and CHT alone (MV 1.8 mm, SD 0.9 mm). CHT + GFC showed a mean value of 1.7 mm (SD 2.3 mm), the blank group a mean value of 1.7 mm (SD 2.3 mm), and the collagen matrix a mean value of 0.3 mm (SD 0.5 mm). The application of collagen matrix + GFC led to the lowest NBH (MV 0.07 mm, SD 0.17 mm). In accordance, the group with the jellyfish collagen matrix as well as the group with collagen matrix + GFC had a significantly lower NBH when compared to the group with collagen powder (*p* = 0.001 and *p* < 0.001; Figure 11). When comparing the blank controls with all treatment groups (MV 1.7 mm, SD 2.3 mm versus MV 1.75 mm, SD 1.6 mm), no significant differences were seen (*p* = 0.96; Figure 10).

## 4. Discussion

A recent review pointed out that surgical treatment of peri-implantitis should be considered in cases of evident bone loss and pocket formation of larger than 5 mm [10]. Chemical, mechanical, and/or laser decontamination of the affected implant surfaces is of high importance for a successful treatment [29]. Especially, the combination of mechanical and chemical removal of the biofilm has been recommended [30]. Even so, there is limited evidence that the peri-implant bone level may be arrested. At the moment, the effectiveness of treating peri-implantitis via different nonregenerative techniques seems to be limited whereas a regenerative approach is considered to be the treatment of choice [10]. Nevertheless, mostly due to a lack of evidence, this is discussed controversially [31]. Schwarz et al., using GBR techniques including collagen membranes, bovine, and equine bone material as well as recombinant human bone morphogenic protein 2 (rhBMP-2), came to the conclusion that predictable results in order to obtain complete defect closure could not be obtained [16]. Other authors could not detect significant differences between the surgical protocols when using GBR techniques as well [12, 32].

In the study at hand, a porcine *in vivo* model was used in order to assess several potential collagen-based techniques for regeneration of bony peri-implant defects. In all groups, an increase in the parameters for bone regeneration was detected. Though, when comparing the results with those obtained with the control group (blank = blood coagulum), neither the addition of different collagen scaffolds nor the combination of collagen scaffolds with growth factors or periodontal ligament stem cells led to a significant enhancement of osseous growth around the implants. Even so, it has to be kept in mind that in all groups a semipermeable membrane was used for GBR purposes. Therefore, it might be concluded that GBR procedures using a collagen membrane, despite of the filling material used, will lead to an enhancement of peri-implant bone growth. This is in accordance to other publications [33–35] whereas Simion and coworkers examined that bovine bone substitute material impregnated with rhPDGF-BB does not benefit from an additional membrane coverage [36]. The rationale for the overlying membrane is to contain blood/scaffolds within the defect, to increase the stability of the wound, and to provide space while excluding soft tissue ingrowth. When using dimensionally stable bone or bone substitute materials, unlike collagen scaffolds, this further stabilization may not be needed [36, 37]. The next potential parameters of influence in all groups were the rough-surface implants with CaP coatings. These have already shown putative advantages such as vertical osteoconductive characteristics in terms of osseous growth during the early healing phases [24, 26]. It was also reported that rough-surface implants or implants with hydroxyapatite surfaces together with membrane techniques (GBR) will lead to a higher degree of bony healing when compared to implants with smooth surfaces [38, 39].

When compared to the jellyfish collagen matrix, the use of a collagen powder consisting of bovine collagen types I, III, and VI showed a significant higher regenerative potential in terms of BIC & NBH. Collagen from marine organisms is thought to be an alternative to collagen of porcine or bovine origin that could be equally effective and saver. Especially, collagen obtained from jellyfish has shown to have stimulatory effects on procollagen synthesis, wound healing, and reduction of scar tissue [40] together with a higher cell variability and viability of fibroblasts when compared to porcine or bovine collagen [41]. Whereas some research was conducted on jellyfish collagen for cartilage tissue engineering yet, experiments on osseous regeneration are still lacking. Nevertheless, as it was detected that jellyfish collagen will stimulate an immune response as well [42], this may counteract the growth of new bone. Further research is needed in order to explain these findings.

The growth factor cocktail used in this study was extracted out of platelet lyophilisate. When combining these factors with collagen carriers, no improved bony peri-implant regeneration was seen. In the past, it was demonstrated that autologous blood preparations containing several growth factors (e.g., PDGF, TGF-b, IGF, VEGF, and bFGF) out of a large number of platelets have a growth-stimulating effect on oral soft and hard tissues including peri-implant bone [43]. Nevertheless, as seen in the study at hand, there are several publications in which none such a beneficial effect could be detected as well [37]. Also, a systematic review on autologous platelet concentrates for postextractional socket healing came to the conclusion that the evidence for positive effects on bone formation is limited [44].

The periodontal ligament is a source of periodontal ligament stem cells (PDLSC) that contribute to bone regrowth after trauma and inflammatory reaction [45]. Therefore, these cells capable of osseous regeneration were seeded into collagen scaffolds around peri-implant bone defects as proposed for treatment of peri-implantitis [20]. Though, in the study at hand, no significant benefits of PDLSC were seen when compared to the other groups. In general, there is limited evidence for using PDLSC in cases of peri-implant bone defects even if the literature supports the findings of the study at hand. In accordance, Park et al. came to the same conclusion when treating bone defects due to peri-implantitis with PDLSC and GBR techniques. Only transplantation of PDLSC transduced with adenoviral vectors containing BMP2 led to a significant enhancement of bony growth when compared to the control group. PDLSC alone could not show superiority [20]. Others demonstrated a significant increase of bone regeneration using PDLSC in peri-implant defects in dogs after 56 days. Though, after 112 days, a time period that was similar to the one used in the study at hand, this significant effect no longer existed, also due to the large standard deviations [46]. A possible reason is that PDLSC have a long-term survival of more than 56 days *in vitro* and may not survive longer when transplanted into an *in vivo* situation as well [47]. In accordance, the effect of PDLSC in bony regeneration may occur earlier. Nevertheless, it needs to be stated that in this study 2D histological specimens were examined only. A further examination of the specimens using 3D techniques such as microCT could obtain additional information.

## 5. Conclusion

GBR using a collagen membrane for coverage of a peri-implant defect together with rough-surface CaP-coated implants will lead to a certain extent of osseous regeneration of the respective defects. The addition of collagen scaffolds together with platelet-derived growth factors or periodontal ligament stem cells will not enhance the osseous regeneration significantly. Though, all results are not predictable and the standard deviation is quite high.
